# Association Between Patent Foramen Ovale and Overt Ischemic Stroke in Children With Sickle Cell Disease

**DOI:** 10.3389/fneur.2021.761443

**Published:** 2021-12-13

**Authors:** Najibah A. Galadanci, Walter Johnson, April Carson, Gerhard Hellemann, Virginia Howard, Julie Kanter

**Affiliations:** ^1^Division of Hematology and Oncology, UAB School of Medicine, University of Alabama at Birmingham, Birmingham, AL, United States; ^2^Department of Pediatrics, UAB School of Medicine, University of Alabama at Birmingham, Birmingham, AL, United States; ^3^Jackson Heart Study, University of Mississipi Medical Center, Jackson, MS, United States; ^4^Department of Biostatistics, School of Public Health, University of Alabama at Birmingham, Birmingham, AL, United States; ^5^Department of Epidemiology, School of Public Health, University of Alabama at Birmingham, Birmingham, AL, United States

**Keywords:** sickle cell disease, children, ischemic stroke, patent foramen ovale, sickle stroke screen

## Abstract

Ischemic stroke is one of the most devastating complications of sickle cell anemia (SCA). Previous studies have shown that intracardiac shunting including patent foramen ovale (PFO) can be a potential risk factor for stroke in children with SCA. This study investigates the association between PFO and overt ischemic stroke in the DISPLACE (Dissemination and Implementation of Stroke Prevention Looking at the Care Environment) study cohort of 5,247 children with SCA of whom 1,414 had at least one clinical non-contrast transthoracic echocardiogram. Presence of PFO was taken from the clinical report. Further, we assessed the association between PFO and other clinical and hemolytic factors in children with SCA such as history of abnormal sickle stroke screen [elevated Transcranial Doppler ultrasound (TCD) velocity] and patient's baseline hemoglobin. In 642 children for whom all data were available, the adjusted odds ratio (OR) for overt stroke was higher in those with PFO but this was not statistically significant (OR: 1.49, 95% CI: 0.20–11.03, *p* = 0.6994). With an OR of 0.85, the study suggested less PFOs in those with abnormal TCD, but this was not statistically significant (95% CI: 0.17–4.25, *p* = 0.8463). Overall, the prevalence of PFO in this large sub study of non-contrast echocardiography amongst children with SCA is much lower than previous smaller studies using bubble contrast echocardiography. Overt stroke was non-statistically more common in children with SCA and PFO, but there was no evidence that PFO was more common in those with abnormal TCD, the most important pediatric sickle stroke screen.

## Introduction

Sickle cell anemia (SCA) is the leading cause of overt ischemic stroke in children (1), with a stroke risk up to 10% per year without any primary stroke prevention strategy applied (2–5). Stroke is one of the most devastating complications in children with SCA leading to high morbidity and mortality (3, 4). Several risk factors have been identified for stroke in children with SCA including anemia, leukocytosis, hypertension, silent infarction, history of acute chest syndrome, and other environmental and genetic factors (4, 6).

Previous studies have shown patent foramen ovale (PFO) to be a significant risk factor for overt ischemic stroke in adults (without SCA) with a prevalence of up to 40% in young adults (7, 8). PFO leads to paradoxical embolization where emboli from the systemic venous circulation pass from the right heart directly into the left heart and eventually the brain without filtration in the lungs (8–10).

Children with SCA have been shown to be at a 4- to 100-fold increased risk for thrombosis compared with the general population (11). SCA is associated with increased activation of coagulation mechanism leading to increased circulating thrombin, platelet activation, increased activation of fibrinolysis and decreased levels of anticoagulant proteins (12–15). In SCA, the chronic anemia and pulmonary arterial hypertension may lead to increased right heart pressure thereby predisposing to right to left shunting and paradoxical embolization; this may predispose children with SCA who have a PFO to increased risk for stroke (16). Previous studies have shown that potential right-to-left shunting through the heart or the lungs, diagnosed with bubble contrast transthoracic echocardiograms, can be a risk factor for stroke in children with SCA (17, 18). Unfortunately, because of the perceived risks of bubble contrast in children with SCA without stroke, most of these studies were limited by their small sample size and the use of control groups of children without SCA (17).

The greatest advancement to date in preventing strokes in children with SCA has been using transcranial doppler ultrasound (TCD) screening (now called a sickle stroke screen) to identify children who are at risk of stroke and institute primary prevention strategy (2). The sickle stroke screen is used to detect stenosis in the distal intracranial portions of the internal carotid artery and the proximal middle cerebral artery MCA as evidence by elevated TCD velocity in the arteries (19). The STOP trial demonstrated that children with elevated TCD velocity >200 cm per second that received regular blood transfusion therapy had a 92% relative risk reduction in the rate of overt strokes when compared with standard therapy. Despite the success shown in the STOP trial, and the subsequent published guidelines, many centers continue to have poor implementation of the recommended annual TCD screening (20–24). In addition, currently it is unclear whether the etiology of strokes in children and adults occurring in the era of sickle stroke screening with TCD are related to alternative stroke mechanisms or implementation failure of the screening system. Preliminary results from the DISPLACE study suggest that the majority of ischemic strokes occurring in the current era are most often occurring in children who have not had a sickle stroke screen/TCD in the preceding 365 days (publication pending). Although causality cannot be assessed in DISPLACE, it does appear that a failure of implementation of sickle stroke screen is in part responsible for these events.

Recently, the DISPLACE (Dissemination and Implementation of Stroke Prevention Looking at the Care Environment) study was funded by National Heart Lung and Blood Institute (NHLBI) to evaluate implementation of TCD screening and assess barriers and facilitators to evidence-based stroke prevention for children with SCA across 28 sites in the USA (25). As part of DISPLACE study, data were collected on children with SCA who were aged 2–16 at the time of the study and included TCD results, MRI results, Echocardiogram information (if done for clinical assessment) as well as laboratory and demographic information. The overall goal of the current study was to investigate the association between PFO and overt ischemic stroke in the DISPLACE cohort. Further, we assessed the association between PFO and other clinical and hemolytic factors in children with SCA such as abnormal TCD, baseline hemoglobin and blood pressure.

## Methods

### Study Population and Design

DISPLACE study retrospectively collected data on 5,247 children with SCA aged between 2 and 16 years from 28 centers in the US including clinical and laboratory assessments from defined study years of 2012–2016 (25). These data included baseline values of heart rate and blood pressure measurements, anthropometric measurements including height and weight and presence or absence of sickle cell disease-related therapies including chronic red cell transfusion (CRCT) and hydroxyurea (HU) therapies. Laboratory data included baseline data on complete blood count including hemoglobin and reticulocyte count.

While the clinical and anthropomorphic data focused were only collected during years 2012–2016, data from radiographic studies were uploaded from birth-present. Thus, Magnetic Resonance Imaging (MRI), TCD, and echocardiogram reports were included if available throughout the lifespan of the individuals. All of these radiographic studies including echocardiograms were ordered by the treating provider for standard of care (not for study-specific inquiry) and were not read centrally. As shown in the practice pattern questionnaire for echocardiography among participating centers in the DISPLACE study, about 50% of the centers order echocardiogram as part of routine care for children with SCD, while the remaining 50% order echo in children only secondary to other clinical circumstances including history of overt stroke, history of silent stroke, asthma, LVH on chest x-ray, murmurs that in non-typical flow murmur.

All echocardiogram studies were performed and interpreted according to the American Society of Echocardiography guidelines (26, 27). The echocardiogram reports collected ranged from 2000 to 2020. There was no central adjudication of the echocardiograms or the reports; the data were only collected as reported. The case report forms for neurologic complications and MRI findings included the options for overt ischemic stroke, silent cerebral infarct, and hemorrhagic stroke. Only the overt ischemic stroke was included in this project.

**Figure 1:**
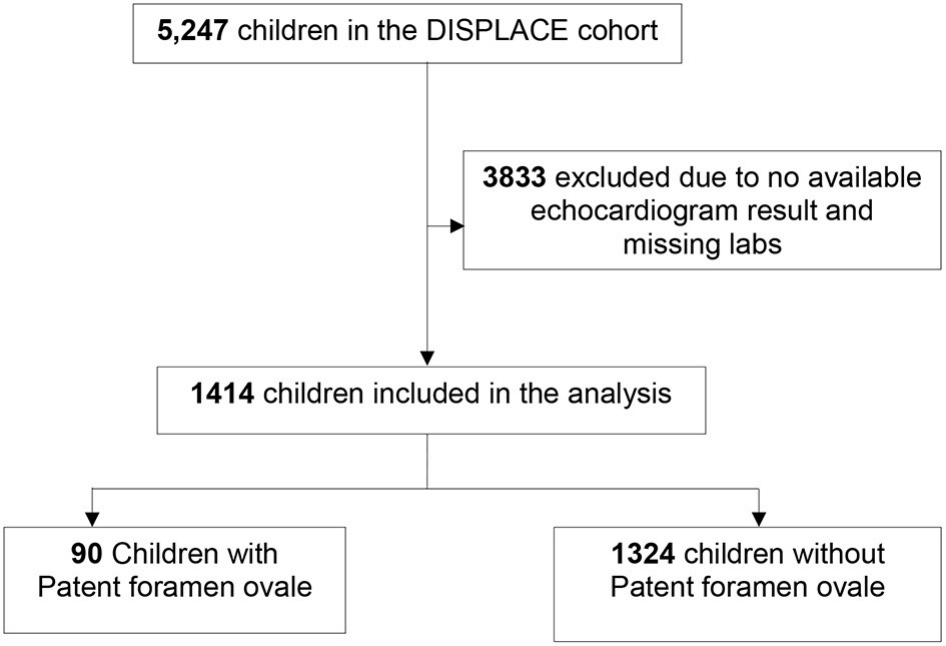
Flow chart depicting the number of DISPLACE study participants included in the analysis. DISPLACE, Dissemination and implementation of stroke prevention looking at the care environment.

The current study was conducted as a cross-sectional analysis of children with SCA who had at least one echocardiogram and one set of laboratory and clinical assessments (during the same calendar year as the echocardiograph). Of the 5,247 children in the DISPLACE database, 3,833 were excluded who did not have an echocardiogram or accompanying lab and clinical assessment resulting in an analysis cohort of 1,414 (Figure 1).

### Institutional Review Board and Data Use Agreements

Institutional Review Board (IRB) approval and data use agreement for the DISPLACE study were obtained at and between each clinical institution and the sponsoring institution using a common protocol (25). All data were de-identified at time of entry and all data were retrospective; thus, consent was not required from individual patients or their families. For the current analysis, we further obtained IRB approval from the University of Alabama at Birmingham to evaluate the de-identified data.

### Variable Definitions

Our primary outcome of interest was the relationship between presence of PFO and presence of overt ischemic stroke. Our secondary outcome is abnormal TCD velocity as recorded into the DISPLACE database. Our predictor variable was PFO defined as present/absent on the echocardiogram report. Our covariates included age in years, sex, systolic blood pressure (SBP), diastolic blood pressure (DBP), hemoglobin level, reticulocyte count and ever or never use of (HU) or CRCT. Selection of our covariates was based on previous knowledge on literature about their importance in the research area (28, 29). All variables were identified from the DISPLACE database.

### Statistical Analysis

Descriptive analysis was carried out to examine the association between PFO and covariates. Median and interquartile range (IQR) were reported for age, hemoglobin level, reticulocyte count, SBP, and DBP, whereas frequencies and percentages were reported for sex, HU, and CRCT. In the bivariate analysis, Kruskal-Wallis test and Cochran-Mantel-Haenszel test were conducted for continuous and categorical variables respectively. To determine the association between PFO and overt ischemic stroke, a logistic regression was fit adjusting for covariates. Using the no PFO as the referent group, crude and adjusted odds ratios (ORs) with their accompanying 95% confidence intervals (CIs) were calculated for the group with PFO. Further, to determine the association between PFO and abnormal TCD velocity, we used a logistic regression and adjusted for potential covariates.

For all analysis, we used complete case analysis to handle missing data, where we include only individuals with data on all variables. Further to ensure our sample is similar in baseline characteristics with the other children in the DISPLACE cohort that were not included in our analysis, we compared the demographics and clinical characteristics of children included in our analysis to the children that were excluded due to the missing echocardiogram results.

All reported *P*-values were 2 sided. Statistical significance was defined as *p* < 0.05. All data analysis were performed using SAS 9.4 (SAS Institute, Cary, NC).

## Results

### Participants' Characteristics at Baseline

The baseline characteristics of the children are shown in table 1. A total of 1,414 children with SCD from DISPLACE were included in the analysis who had completed echocardiograms in the DISPLACE database. All of the children had SCA (HbSS or HbSB0 disease). All children had at least one documented echocardiogram entered in the DISPLACE database, one clinical assessment result and one complete blood count laboratory result. Of the 1,414 children, 6.3% had PFO noted by echocardiogram (Table 1).

**Table 1:** Demographics and clinical characteristics for children with sickle cell anemia comparing children with PFO to those without.

	***N***	**PFO**	**NO PFO**	***P*** **-value**
Number	1,414	90	1,324	
Age (years)	1,414	4.8 ± 5.3	8.7 ± 46	<0.0001
Sex^*^, ***n*** **(%)**				0.2967
Male	725	41 (46.1)	684 (51.8)	
Female	685	48 (53.9)	637 (48.2)	
Hemoglobin (g/dl)^†^	950	8.9 (8.0, 9.9)	8.6 (7.8, 9.6)	0.3624
Reticulocyte (per 1,000)^†^	437	326 (137.8, 407.5)	283.0 (158.0, 395.0)	0.8371
Systolic BP (mmHg)^†^	642	107 (96, 117)	109 (101, 116)	0.2861
Diastolic BP (mmHg)^†^	642	64 (53, 68)	62 (56, 68)	0.9791
Abnormal TCD, *n* (%)	142	133 (10.5)	9 (10.0)	
History of stroke, *n* (%)	102	97 (7.3)	5 (5.6)	
**Therapy group**, ***n*** **(%)**				0.0960
No therapy	617	51 (58.6)	566 (46.6)	
Blood trans	190	10 (11.5)	180 (14.8)	
Hydroxyurea	494	26 (29.8)	468 (38.5)	

*BP, Blood pressure (mmHg); Blood trans, Chronic red cell transfusion; PFO, Patent foramen ovale*.

† *Variables are reported as median (interquartile range)*.

* *Variables are reported as the frequency and percent relative to the row attribute*.

*P-values from Row mean zero scores differ using Cochran-Mantel-Haenszel test for categorical and ttest or Wilcoxon rank sum test for continuous variables*.

The mean age of the children who had PFO was 4.8 years and this was significantly lower than those without PFO (8.7years) (*p* < 0.0001). There were no differences in sex, systolic or diastolic blood pressure, hemoglobin, and/or reticulocyte count between those with PFO and those without PFO (Table 1). A lower proportion of children with PFO had been treated on CRCT compared to children without PFO, while a higher proportion of children with PFO had been treated on HU compared to children without, but this difference did not reach statistical significance (*p* = 0.0516). About 29% of children with PFO were on HU while only about 10% of children with PFO were on CRCT.

### Association Between PFO and Overt Ischemic Stroke in Children With SCA

A total of 102 (7.2%) of the 1,414 children included in the analysis had an overt ischemic stroke. Out of this 5 (5.6%) had PFO. The odds of stroke was higher in those with PFO but this was not statistically significant [OR: 1.49, 95% CI: 0.20–11.03, *p* = 0.6994; Table 2].

**Table 2:** Crude and adjusted odds ratios^**^ and 95% Confidence intervals (95% CI) for the association between patent foramen ovale and ischemic stroke.

	**Total number**	**++No PFO**	**PFO**
Patients	1,414	1,324	90
Patients with ischemic stroke (%)	102	97 (7.3)	5 (5.6)
Crude odds ratio (95% CI)	1,414	1.0 (ref)	0.74 (0.30–1.88)
^*^Adjusted odds ratio (95% CI)	642	1.0 (ref)	1.49 (0.20–11.03)

*CI, Confidence interval; PFO, Patent foramen ovale*.

** *Logistic regression was used to calculate odds ratio comparing children with PFO to those without PFO*

* *adjusted for age, sex, systolic blood pressure, diastolic blood pressure. ++The reference group is the group with no PFO. The sample size used for the adjusted model dropped from 1,414 to 642 due to missing values in some of the covariates included in the model*.

### Association Between PFO and Abnormal TCD in Children With SCA

A total of 142 (10.0%) of the 1,414 included in the analysis had abnormal TCD. Out of this, only 9 (10%) had PFO. There is no evidence that the odds of having abnormal TCD is higher among those with PFO compared to those without even after adjusting for potential covariates (OR: 0.85, 95% CI: 0.17–4.25, *p* = 0.8463; Table 3).

**Table 3:** Crude and adjusted odds ratios^**^ and 95% Confidence intervals (95% CI) for the association between patent foramen ovale and abnormal TCD.

	**Total number**	**++No PFO**	**PFO**
Patients	1,414	1,324	90
Patients with abnormal TCD velocity (%)	142	133 (10.5)	9 (10.0)
Crude odds ratio (95% CI)	1,414	1.00 (ref)	0.99 (0.49–2.03)
^*^Adjusted odds ratio (95% CI)	642	1.00 (ref)	0.85 (0.17–4.25)

*N (%), Number (percent); CI, Confidence interval; PFO, Patent foramen ovale; TCD, transcranial doppler ultrasound velocity*.

** *Logistic regression was used to calculate odds ratio comparing children with PFO to those without PFO*

* *adjusted for age, sex, systolic blood pressure, diastolic blood pressure. ++The reference group is the group with no PFO. The sample size used for the adjusted model dropped from 1,414 to 642 due to missing values in some of the covariates included in the model*.

## Discussion

In the current crossectional analysis of a large cohort of children with SCA, we found a lower than expected prevalence of PFO as reported in the routine, presumably all non-contrast, transthoracic echocardiograms. Only 6% of the children with SCA were found to have PFO on routine echocardiogram.

Our results did not support the hypothesis that PFO is associated with overt ischemic stroke in children with SCA as only ~5% of children with PFO had overt ischemic stroke. In contrast to this study, previous studies have reported consistently higher prevalence of PFO in the general population (30) as well as in children with SCA. In a meta-analysis, Mattle et al. showed an increased risk of stroke among adults with PFO compared to those without (30). One study, in which transthoracic and/or trans esophageal echocardiography was used in more than three quarters of children with stroke, found the prevalence of PFO to be low (31). However there is evidence for a higher prevalence of right-to-left shunting, including at atrial level, in children with stroke undergoing bubble contrast, e.g., with transthoracic echocardiography or transcranial Doppler (cTCD) (32, 33); this is considered particularly important in those without vasculopathy (32). Similarly, previous studies have reported an increased risk of stroke among adults and children with SCA and PFO (16, 17, 34). In a study by Dowling et al. they found a significantly higher prevalence of right to left shunting in children with SCA compared to those without SCA. Importantly, this study included any identified Right to Left shunting as the target variable including but not limited to PFO (16). Additionally, they also found that those with any potential right to left shunting had a greater odds of being in the group of children with SCA and stroke compared to children without SCD. There were notable differences between their study population and their target variable and ours which may have contributed to the inconsistencies in the results. We included a control group of children with SCA but without stroke, while they had a control group of children without SCA or stroke.

Considering the pathophysiology of stroke in SCA, our results showed that even though PFO may be a potential risk factor for stroke in the general population, in children with SCA, other risk factors seem to play a more important role in the pathogenesis of stroke than the PFO. The presence of PFO should not be considered to be confirmatory evidence of paradoxical embolic stroke in children with SCA and overt ischemic stroke. As shown in a review of echocardiogram features and paradoxical emboli by Aggeli et al. even among the general population, the presence of PFO does not necessarily confirm the diagnosis of paradoxical embolic stroke in a patient with an overt ischemic stroke. The patient's clinical history, brain, cerebrovascular, and cardiovascular imaging should be reviewed in detail to determine whether embolic stroke is a possibility and if so, candidate sources including carotid or vertebral vasculopathy as well as right-to-left shunting (8). Further, in another review, Aggeli et al. showed that even though PFO may increase the risk of occurrence of an embolic stroke, there were not enough data to determine whether PFO closure or antiplatelet drugs prevented recurrence in these patients (35).

In the current study we found no significant association between PFO and abnormal TCD velocity. Approximately 10% of the children with PFO had abnormal TCD on their sickle stroke screen. Considering abnormal TCD velocity is the most important surrogate use to identify those at increased risk of overt ischemic stroke in children with SCA (19, 36, 37), our hypothesis was that children with SCA with abnormal TCD velocities will have higher prevalence of PFOs compared to those with normal TCD velocities. However, this was not supported by our findings.

Age was found to be significantly associated with PFO. The median age for children with PFO was significantly lower than that of children without PFO. This is expected and consistent with findings from previous studies showing that the majority of PFOs close during infancy, and for some the closure may be gradual during childhood (38). Some studies reported complete anatomic closure in up to 70–75% of adults (39).

In the present study, hemolytic factors including hemoglobin level and reticulocyte count were not significantly associated with presence of PFO. Although there is limited data on the relationship between hemolytic factors and PFO, Kucukal et al. showed that increased red cell adhesion markers correlate strongly with hemolytic markers including hemoglobin level and reticulocyte count and a history of intrapulmonary right to left shunts (40).

### Limitations of the Study

As expected with a retrospective crossectional study design, our study has limitations. DISPLACE is a real-world evaluation of current practice in clinical centers in the US. All echocardiograms were performed according to standard of care for the institution. The reports collected from each center were used according to how they were reported from the center with no additional review conducted as part of this study. PFO was defined as present/absent on the echocardiogram report. Not all children had a bubble study to determine the presence of PFO. We had a low number of events compared to what was reported in previous studies. Additionally, we used only available data and therefore were not able to include echocardiograms with reasonable amount of missing data and DISPLACE participants who do not have echo results. Therefore, in the adjusted models for the logistic regression, the number of children included was only 45% of the full cohort To ensure our study population did not differ from the original DISPLACE study, we compared the baseline characteristics of the participants included in our analysis to the general DISPLACE participants and we found no significant difference in the baseline demographic and laboratory characteristics between the two populations (Supplementary Table 1).

The strengths of our study include the use of a large national population sample of children with SCA from 28 sites across the US, which therefore facilitates the generalizability of our findings. To our knowledge, this is one of the largest studies investigating stroke and PFO in sickle cell disease. In addition, our study used a comparison group of children with SCA without PFO and therefore making the two groups more comparable in terms of baseline characteristics.

## Conclusion

Our study showed a much lower prevalence of PFO among children with SCA than previously published. We found no increased risk of stroke or abnormal TCD among those with PFO compared to those without PFO in children with SCA. This could be due to the fact that overall DISPLACE recorded a much lower frequency of abnormal TCD than reported in older studies (25). We believe that even though PFO may be associated with increased risk of overt ischemic stroke in the general population, in children with SCA, other known risk factors including anemia, hypertension, silent infarction and history of acute chest syndrome, likely play a more important role. Contrast echocardiography or transcranial Doppler may be considered in patients with SCA of any age who have had an overt stroke and have no evidence of vasculopathy on TCD or MRA. If treatment, e.g., closure or antiplatelet therapy, is standard of care for people with cryptogenic stroke and right-to left shunting, larger prospective studies in SCA should focus on diagnosis or exclusion of right to left shunting at cardiac or pulmonary level as a risk factor for overt and silent stroke.

## Data Availability Statement

The datasets presented in this article are not readily available because of privacy reasons. However, aggregated datasets may be shared. Requests to access the datasets should be directed to Julie Kanter, jkanter@uabmc.edu.

## Ethics Statement

The studies involving human participants were reviewed and approved by University of Alabama at Birmingham Institutional Review Board. Written informed consent to participate in this study was provided by the participants' legal guardian/next of kin.

## Author Contributions

NG, WJ, VH, and JK designed the study. AC, GH, and VH wrote and reviewed the manuscript. All authors contributed to the article and approved the submitted version.

## Funding

This study was supported by the grant 5R01HL133896-04 to JK from NIH and Pre-doctoral grant 20PRE35210531 to NG from AHA.

## Conflict of Interest

The authors declare that the research was conducted in the absence of any commercial or financial relationships that could be construed as a potential conflict of interest.

## Publisher's Note

All claims expressed in this article are solely those of the authors and do not necessarily represent those of their affiliated organizations, or those of the publisher, the editors and the reviewers. Any product that may be evaluated in this article, or claim that may be made by its manufacturer, is not guaranteed or endorsed by the publisher.
