# Supplementary material for: Association Between Patent Foramen Ovale and Overt Ischemic Stroke in Children With Sickle Cell Disease
Source: Front Neurol. 2021 Dec 13;12:761443. doi: 10.3389/fneur.2021.761443 (PMC8710657; doi:10.3389/fneur.2021.761443)
Supplement: Supplementary file 1 [file Table_1.docx]

**Supplemental Table 1: Baseline demographic, clinical and laboratory characteristics of the DISPLACE cohort comparing children who were included in the Echocardiographic study and those excluded.**

| **Variable** | **Number** | **Echocardiogram** | **No echocardiogram** | **P value** |
| --- | --- | --- | --- | --- |
| **Number (%)** | 5428 | 1412 (26.0) | 4016 (73.9) |  |
| **Age^#^** | 3480 | 8.5±4.5 | 5.8±4.5 | **<0.0001** |
| **Sex, n(%)*** |  |  |  | **0.2886** |
| Male | 2724 | 725 (51.4) | 1999 (49.8) |  |
| Female | 2702 | 685 (48.6) | 685 (48.5) |  |
| **Race, n(%)*** |  |  |  | **0.3325** |
| Black | 5142 | 1332 (95.7) | 3810 (95.0) |  |
| White | 67 | 21(1.5) | 46 (1.2) |  |
| Others | 16 | 1(0.1) | 15(0.4) |  |
| Unknown | 194 | 53(3.8) | 141(3.5) |  |
| **Insurance, n (%)^*^** |  |  |  | **0.4758** |
| Medicaid | 2498 | 756 (71.8) | 1742 (70.5) |  |
| Others | 973 | 285(27.1) | 688(27.9) |  |
| None | 52 | 12 (1.1) | 40 (1.6) |  |
| **Hemoglobin (g/dl)ⴕ** | 3258 | 8.7 (7.8, 9.6) | 8.6 (7.8, 9.5) | **0.3598** |
| **Reticulocyte count (per 1000)ⴕ** | 2011 | 297.0 (194.4, 408.0) | 295.0 (191.0, 349.0) | **0.4696** |

Abbreviations: n(%)- number(percent), ⴕ value reported is median and Interquartile range, # value reported is mean ± 2standard deviation, * value reported is frequency and percentage of total.
